# Macroscale Plasmonic Substrates for Highly Sensitive Surface-Enhanced Raman Scattering**

**DOI:** 10.1002/anie.201302285

**Published:** 2013-04-29

**Authors:** Maria Alba, Nicolas Pazos-Perez, Belén Vaz, Pilar Formentin, Moritz Tebbe, Miguel A Correa-Duarte, Pedro Granero, Josep Ferré-Borrull, Rosana Alvarez, Josep Pallares, Andreas Fery, Angel R de Lera, Lluis F Marsal, Ramón A Alvarez-Puebla

**Affiliations:** Department of Electronic Engineering, Universitat Rovira i Virgili, Avda. Països Catalans26, 43007 Tarragona (Spain) E-mail: lluis.marsal@urv.catramon.alvarez@urv.cat; Department of Physical Chemistry II, University of Bayreuth, Universitätsstrasse 30Bayreuth 95440 (Germany) E-mail: andreas.fery@uni-bayreuth.de; Department of Organic and Physical Chemistry, University of Vigo36310 Vigo (Spain) E-mail: qolera@uvigo.es; Department of ICREA (Catalonian Institution for Research and Advanced Studies), Avda. Lluís CompanysBarcelona, 08010 (Spain) and Center for Chemical Technology of Catalonia, Edifici N5, Campus de SesceladesCarrer de Marcel⋅lí Domingo s/n 43007 Tarragona (Spain)

**Keywords:** macroscale arrays, nanoparticles, optical sensors, plasmonic films, surface-enhanced Raman scattering

The fabrication of macroscale optical materials from plasmonic nanoscale building blocks is the focus of much current multidisciplinary research. In these macromaterials, the nanoscale properties are preserved, and new (metamaterial) properties are generated as a direct result of the interaction of their ordered constituents.^1^ These macroscale plasmonic assemblies have found application in a myriad of fields, including nanophotonics, nonlinear optics, and optical sensing.^2^ Owing to their specific requirements in terms of size and shape, their fabrication is not trivial and was until recently restricted to the use of lithographic techniques, especially those based on electron- or ion-beam patterning.^3^ However, these techniques are not only expensive, time-consuming, and demanding but are also restricted to small simple and solid geometries, which are good for proof of concepts but less suitable for real-life applications. Approaches based on colloidal chemistry are gaining relevance as an alternative. During the past few years, several examples of the fabrication of organized particles have been reported, including the preparation of complex colloidal particles^4^ and the use of preformed colloids to create large crystalline organized entities known as supercrystals.^5^ The latter approach provides optical platforms with unprecedented plasmonic properties that can be exploited for the design of cheap ultrasensitive and ultrafast sensors with surface-enhanced Raman scattering (SERS)^6^ spectroscopy as the transducer.

We report a new template-assisted method based on the stamping of colloidal particles for the large-area fabrication of organized pyramidal supercrystal periodical arrays. The extraordinary optical activity of these pyramidal supercrystals is demonstrated both theoretically and experimentally. The plasmonic platform is then exploited for the development of a handheld reversible SERS sensor for the live monitoring of carbon monoxide in the atmosphere. CO is a ubiquitous colorless, odorless, and tasteless gas produced during incomplete combustion (during tobacco smoking or in car engines and furnaces) which poses a potentially fatal threat to human health.

The method used for the preparation of the nanostructured pyramidal arrays is illustrated in the Figure 1. First, inverted pyramidal templates were prepared by direct laser writing lithography on oxidized p-type silicon wafers, followed by a chemical etching process (see the Supporting Information for details). This method yields periodically patterned surfaces with homogenous inverted pyramids with dimensions that can be tuned from 1 to 10 μm as a function of the etching time (see Figure S3 in the Supporting Information). In this study we chose a period of 8 μm to generate pyramids with sides of 4.5 μm and a height of 3.3 μm (Figure 1 B). This size enables the preparation of a truly macroscale nanostructured material that can be observed with a conventional optical microscope and permits detailed characterization of the optical-enhancing properties of the nanostructures. Prior to the deposition of the nanoparticles (NPs), the surfaces were cleaned with an oxygen plasma. A concentrated solution of gold NPs was then cast on the template, allowed to dry, and then transferred to the surface to yield a periodic array of square pyramids (Figure 1 C; see the Supporting Information for details) derived from the compact packing of plasmonic particles (Figure 1 D; see also Figures S4–S6). Although the film was transferred to many surfaces, including glass, silicon wafers, and double-sided tape, we describe herein the studies carried out on versatile and flexible poly(dimethylsiloxane) films (1 mm thick). AFM characterization of the film (see Figure S7) showed nanoparticle pyramids with high homogeneity in all directions, with side lengths of 4.4 μm and a height of 3.0 μm.

**Figure 1:**
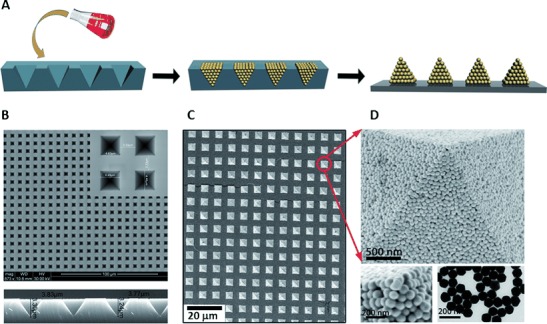
A) Schematic representation of the fabrication of the macroscale nanostructured film. B) Empty inverse pyramidal lithographic surfaces used as templates. C) SEM image of the macroscale plasmonic film after stamping. D) High-resolution SEM images of the pyramids and TEM image of the gold nanoparticle building blocks.

Close observation of single pyramids (Figure 1 D; see also Figure S4) offers some insight into their structure, which in combination with the results of other studies allows us to propose a mechanism of formation. A monodisperse collection of spherical, organic-ligand-coated (in this case, with cetyltrimethylammonium bromide, CTAB) nanocrystals is expected to form a face-centered cubic lattice in a confined volume.^5^, ^7^ When dispersed in a solvent, the nanocrystals experience short-range steric repulsions.^8^ However, when the nanoparticles are compressed together and their total density exceeds a critical value, the nanocrystals spontaneously assemble into a supercrystal. This ordering transition is driven by entropy. With negligible energetic interactions between nanocrystals, only the excluded volume of each particle matters, and the structure with the highest entropy is favored. The nanocrystals in a “dry” superlattice are held together by strong cohesive interactions between neighboring ligands and nanocrystals. Also, the repulsions between the hydrophobic supercrystal and the hydrophilic walls of the template favor the impression of the nanostructured features when the template is stamped against a surface.^9^ Prior to its optical characterization, the film was cleaned with an oxygen plasma to remove CTAB and favor the contacts between the gold surfaces and the analytes used for SERS. This cleaning process does not affect the geometry of the pyramids (see Figure S8), as previously demonstrated with other supercrystals.5a,c

The formation of pyramidal supercrystals leads to strong plasmon coupling between the AuNPs. Figure 2 A shows the experimental localized surface plasmon resonances (LSPRs) of the AuNPs in solution and after assembly into macroscale pyramids. Noninteracting nanoparticles exhibited a maximum at 540 nm characteristic of their dipolar plasmon mode. After assembly, the dipolar mode was red-shifted to 590 nm, which indicates a significant interparticle coupling. The supercrystals also showed a stronger LSPR contribution in the near-infrared (NIR) region, between 700 and 950 nm. To clarify the nature of this broad feature, we performed finite element method (FEM) calculations with the COMSOL Multiphysics package (see the Supporting Information for details). Tight-binding analysis of the plasmon resonances in the supercrystal indicated the accumulation of an electric near field at the surface and the apex of the pyramid (inset in Figure 2 A; see also Figure S9). This effect is of central importance for the next generation of rapid and portable optical sensors. As a proof of concept, a diluted solution of 1-naphthalenethiol (1NAT; 10 μL, 10^−8^ m) was spin coated on 1 cm^2^ of the pyramid film, and the surfaces were studied with an NIR laser line (785 nm). Although extremely strong SERS signals were acquired for 1NAT (Figure 2 B) at all points, SERS mapping (Figure 2 C) with a very low laser power at the sample (10 μW, with an acquisition time of 10 ms) clearly indicated a significant signal concentration at the apex of the pyramids. This effect was confirmed by high-resolution confocal SERS measurements on a single pyramid with spatial-resolution steps of 500 nm (Figure 2 D).

**Figure 2:**
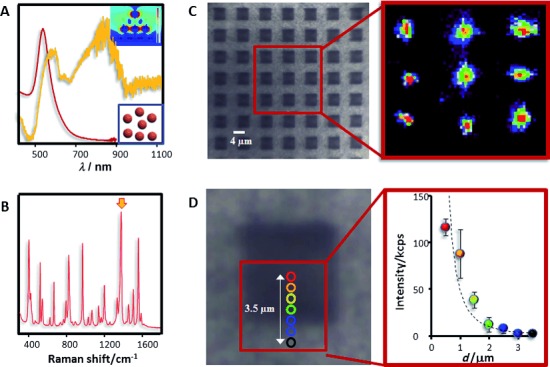
A) Normalized UV/Vis/NIR spectra of the gold nanoparticles in solution (red) and after their impression into plasmonic films (yellow). Top inset: distribution of the near electric field in a pyramid composed of particles. B) Representative SERS spectrum of 1-naphthalenethiol on the pyramid film. The spectrum is characterized by ring stretching (1553, 1503, and 1368 cm^−1^), CH bending (1197 cm^−1^), ring breathing (968 and 822 cm^−1^), ring deformation (792, 664, 539, and 517 cm^−1^), and C–S stretching (389 cm^−1^). C) Optical image and SERS imaging of the band highlighted in B with an arrow. The SERS image shows enhancement mapping with higher signals concentrated around the center of the pyramids. D) Optical image of one pyramidal structure and comparison of the intensities provided by different areas of the plasmonic film. All spectra were acquired with a benchtop high-resolution confocal Raman microscope (acquisition conditions: *λ*_ex_=785 nm, 10 ms, power at the sample: 10 μW, spatial resolution: 500 nm).

Although the remarkable optical activity of these macroscale plasmonic platforms makes them exceptional candidates for academic applications, such as single-molecule detection^10^ (see Figure S10), in this study we designed an ultrafast and reversible optical sensor for the monitoring of carbon monoxide (CO) with an inexpensive handheld Raman spectrometer (see Figure S11). Optical nanoantennas have already been reported for the detection of analytes in solution^11^ and of inorganic gases.^12^ In the case of inorganic gases, these approaches rely on the fabrication of segregated alloys containing silver or gold as the optically active material and another metal (usually platinum or palladium) as the capture material. However, the deposition of the trapping metal not only hinder the adsorption of the gas onto the optical material and lead to the corresponding decrease in sensitivity, but it is also not reversible. Once the gas is adsorbed on the metal, it does not desorb; thus, the sensor can only be used once. This strategy may be suitable for the detection of exotic gases, such as chemical warfare agents, but not for the effective monitoring of a toxic but ubiquitous gas, such as CO. In this case, the sensor should not only be quantitative, sensitive, and fast, but also operate reversibly so that it can inform the user when the concentration of the toxic species is above or below the toxic range.

An alternative approach that fulfills these requirements is the monitoring of the vibrational changes induced on a SERS highly active secondary probe directly bound to the sensor, before and after interaction with the target.^13^ The good affinity and reversible binding of myoglobin and hemoglobin to oxygen and carbon monoxide are known and in fact form the basis of the toxicity of carbon monoxide. Unfortunately, the use of proteins in SERS is not the best solution, as these biopolymers are usually characterized by poor SERS cross-sections. Alternatively, it is possible to functionalize the plasmonic surfaces with an iron porphyrin, the genuine factor responsible for selective and reversible gas capture in blood with binding affinities even higher than those of the proteins.^14^ However, to force a perpendicular orientation of these molecules on the pyramids, as required for the efficient capture of atmospheric gases, the introduction of a single thiol group at just one location of the porphyrin periphery was necessary. Thus, 5-[(triisopropylsilyl)thio]-10,20-diphenylporphyrin (TDPP) was synthesized, complexed with Fe^II^, and self-assembled onto the gold pyramids (see the Supporting Information for details).

Figure 3 B shows the optical response of TDPP before and after iron coordination. Although both spectra showed the characteristic Soret and Q bands, the Soret band was red-shifted from 420 to 442 nm upon formation of the iron porphyrin as a result of the distinct electronic environment brought about by the metal coordinated to the porphyrin center, and the four Q bands in the visible region collapsed into essentially two bands owing to the higher *D*_4*h*_ symmetry of the TDPP–Fe complex. These two features clearly indicate the successful coordination of the metal. Comparison of the Raman and SERS spectra of TDPP (see Figure S13) showed an intensification of the modes corresponding to ring stretching and in-plane deformations and indicated that the molecular plane of TDPP is perpendicular to the plasmonic surface, in full agreement with the surface selection rules.^15^ This result is also consistent with the preparation method, in which a dilute solution of the molecular probe was cast on the plasma-cleaned surfaces of the pyramids. SERS spectra for free and metal-coordinated TDPP (Figure 3 C) were also consistent with the electronic spectra. Although bands directly related to the iron atom can be clearly seen below 700 cm^−1^,^16^ the most remarkable differences are found for the chromophore (i.e., band joining and shifting) as a consequence of the constraints induced by the coordination of Fe^II^.^16^ Notably, after the complexation of TDPP–Fe with CO, several characteristic changes were observed (highlighted with arrows in Figure 3 C). In fact, by following these spectral changes for TDPP–Fe before and after CO complexation it is possible to obtain quantitative information on the amount of CO present in the environment at a given moment.

**Figure 3:**
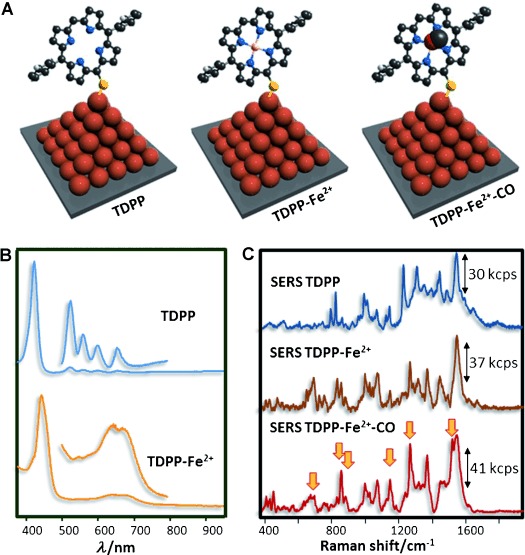
A) Schematic representation of the CO sensor composed of a macroscale plasmonic film and an iron porphyrin (TDPP). B) UV/Vis/NIR spectra of the porphyrin before and after complexation with Fe^II^. C) SERS spectra of the free porphyrin, the porphyrin coordinated to iron, and the iron porphyrin complexed with CO. The spectra are characterized by ring stretching (1549, 1490, 1444, 1370, and 1320 cm^−1^), CCN bending (1268 and 1240 cm^−1^), CCH bending (1146 and 1070 cm^−1^), ring breathing (1026 and 999 cm^−1^), ring deformation (880 and 857 cm^−1^), and N–Fe stretching (591, 569, 506, and 420 cm^−1^). Arrows in the red spectrum highlight the spectral changes after CO complexation. All spectra were acquired with a handheld Raman macrosystem (acquisition conditions: *λ*_ex_=785 nm, 1 s, power at the sample: 1 mW, spatial resolution: 1 mm). Double-headed arrows indicate the intensity.

The detection limits and ranges of these indirect sensors depend strictly on the amount of sensing molecules on the plasmonic surfaces. The amount of molecules required for a good SERS signal depends also on the SERS cross-section of the probe molecule. TDPP–Fe, as any porphyrin, is characterized by its high SERS cross-section, which enables single-molecule detection to be reached.^17^ Thus, in principle, the use of this biointerlayer mimic would enable the detection of CO in the single-molecule regime. In practice, this sensitivity is unnecessary. Exposure to 500 ppm of CO for 1 h can be fatal, whereas CO at a concentration of 100 ppm causes headaches and drowsiness, and 50 ppm of CO induces deterioration of motor skills; however, at CO concentrations below 40 ppm, no symptoms have been reported.^18^ Thus, to set a detection-limit range between 1 and 100 ppm, we explored the effects of different amounts of TDPP–Fe molecules on the pyramid film. Optimal results were obtained by the spin coating of 10 μL of a 3×10^−6^ m solution of TDPP–Fe per square centimeter of surface. Under a confocal Raman microscope, this concentration yielded a very high SERS signal with very low power at the sample (1 μW) and an acquisition time of 10 ms owing to the extraordinary optical activity of the pyramids. These parameters enable the use of the portable handheld Raman system for macroscopic measurements. Deconvolution of the bands at 1516 and 1552 cm^−1^ (Figure 4 A)^19^ and plotting of the band-area ratio against the CO concentration gave a linear correlation, with an impressive *R*^2^ value of 0.9917 (Figure 4 B). This result demonstrates the quantitative nature of this method of analysis. Furthermore, under normal atmospheric conditions, the signal decreases over time (Figure 4 C) owing to the competition between CO and O_2_. After about 20 min, the signal could not be observed; however, after only 5 min, the signal decreased below 20 ppm CO: a tolerable level for humans. With this information in mind, we designed several experiments on the same substrate for the evaluation of the reversibility of the sensor. The active sensor was always recovered in less than 5 min after exposure to air (Figure 4 D); it could therefore be used for continuous monitoring of this gas in the environment.

**Figure 4:**
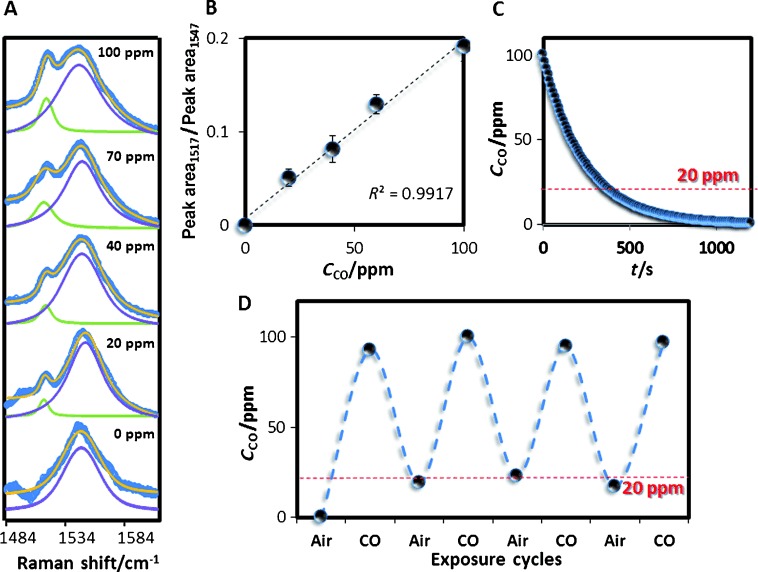
A) Normalized deconvolution of bands 1547 and 1517 cm^−1^ (both of which are due to pyrrole-ring stretching) on the basis of an assumed Lorentzian shape, whereby the band position and the full width at half-maximum are fixed. Blue: experimental spectrum; yellow: spectrum resulting from the addition of peak 1 (green) to peak 2 (purple). B) Linear plot of the area ratio of the peaks at 1517 and 1547 cm^−1^ as a function of CO concentration. Error bars represent the standard deviation for five replicated experiments. C) Signal decay due to the displacement of CO by O_2_ as a function of time. D) Sensor reversibility during several cycles of exposure to CO and air.

In summary, we have demonstrated the feasibility of patterning homogeneous macroscale nanoparticle architectures over large areas. Owing to the interaction of the nanoparticles, the pyramids show a considerable plasmon accumulation on their surfaces and, in particular, at the tips. These plasmonic macrosubstrates were exploited for the fabrication of a reversible and portable optical sensor for CO.

## Supplementary material

As a service to our authors and readers, this journal provides supporting information supplied by the authors. Such materials are peer reviewed and may be re-organized for online delivery, but are not copy-edited or typeset. Technical support issues arising from supporting information (other than missing files) should be addressed to the authors.
