# Supplementary material for: Macroscale Plasmonic Substrates for Highly Sensitive Surface-Enhanced Raman Scattering
Source: Angew Chem Int Ed Engl. 2013 Apr 29;52(25):6459–63. doi: 10.1002/anie.201302285 (PMC3749443; doi:10.1002/anie.201302285)
Supplement: Supplementary file 1 [file anie0052-6459-SD1.pdf]

Angewandte Chemie
Eine Zeitschrift der Gesellschaft Deutscher Chemiker

Supporting Information
© Wiley-VCH 2013
69451 Weinheim, Germany

Macroscale Plasmonic Substrates for Highly Sensitive Surface-Enhanced Raman Scattering**

Maria Alba, Nicolas Pazos-Perez, Belén Vaz, Pilar Formentin, Moritz Tebbe, Miguel A. Correa-Duarte, Pedro Granero, Josep Ferré-Borrull, Rosana Alvarez, Josep Pallares, Andreas Fery, Angel R. de Lera,* Lluis F. Marsal,* and Ramón A. Alvarez-Puebla**

anie_201302285_sm_miscellaneous_information.pdf

Materials and Methods:

Inverted pyramid template fabrication: p-type silicon wafers were oxidized in dry O_2 at 1000 °C for 15 min, in order to grow a thin silicon dioxide film (SiO_2). The oxide film only serves as a mask and as a protective layer in the subsequent anisotropic etch of the silicon in tetramethyl ammonium hydroxide (TMAH, Aldrich) solution. The oxidation step cannot be avoided as the normal resists used in the lithography are easily dissolved by TMAH. Direct laser writing lithography, was used to define the arrangement and period of the resulting pore lattice. A positive photoresist AZ 1505 with a developer AZ 726 metal ion free (Micro Chemicals) were used in the lithography. Following, the lithographic pattern was then transferred into the oxide layer by etching the silicon oxide in buffered hydrofluoric acid (BHF, Aldrich). The used BHF etching mixture (Ammonium fluoride etching mixture HF (6 %) and NH4F (35 %), Honeywell Specialty Chemicals Seelze GmbH) has an etching rate of about 680 Å/min. Therefore, a few seconds are needed to etch the oxide which can result in an over-etching if very thin oxide layers are used. Next, the photoresist was removed and the silicon wafers were immersed in 8 % TMAH solutions at 80 °C temperature for 7-9 min, in order to prepare the defect sites on the wafer surface. The TMAH etch is an anisotropic process; the resulting structure is a lattice of inverted pyramids. After the TMAH etch, the oxide layer is no longer needed so was removed with a HF 5 % solution.

Synthesis of gold nanoparticles: Highly monodisperse spherical gold nanoparticles (~70 nm) were prepared using a previously reported seed mediated approach [1] similar to that used for the production of gold nanorods [2, 3]. Briefly, a seed solution was prepared by mixing an aqueous solution (20 mL) containing $HAuCl_4$ (2.5 10^{-4} M, Aldrich) and trisodium sodium citrate (2.5 10^{-4} M, Aldrich). The mixture was vigorously stirring meanwhile $NaBH_4$ (0.1 M, 600 μL, Aldrich) was added. A fast colour change into red was observed after the $NaBH_4$ addition indicating the formation of the gold particles. The seeds were left under stirring for 1 h to allow the $NaBH_4$ decomposition and with the open bottle to avoid over pressure. Next, a grow solution was prepared by dissolving cetyltrimethylammonium bromide (CTAB, Merck) in milli-Q water (0.1 M, 250 mL) and 0.3 mg per kg of CTAB of potassium iodide (Aldrich). Followed by the addition of $HAuCl_4$ (0.103 M, 1271 μL) and ascorbic acid (0.1 M, 2088 μL, Aldrich).

After each addition the bottle was energetically agitated. After that, 500 µL of the seeds solution were added to the growth solution and was vigorously stirred. The bottle was left undisturbed at 28 °C for 48 h. Afterwards a small amount of sediment is observed in the bottom of the flask. Carefully, the supernatant is collected and the precipitate discarded. The gold particles (200 mL) were concentrated by centrifugation (4 x 7000 rpm, 15 min) to a total volume of 2 mL. Then, the CTAB concentration was decreased from 0.1 M to a final concentration of 0.006 M by diluting 100 µL of the concentrated Au solution with water (1.7 mL). Finally a centrifugation (6000 rpm, 10 min) step was done to achieve a final gold concentration of 3.5 mg/mL.

Nanoparticle Assembly: The preparation of the macroscaled nanostructured pyramidal arrays was achieved as follows. First, the inverted pyramid templates were treated with an oxygen plasma (O_2 0.2 mbar, at 0.1 kW and 2 min in a Flecto10, from Plasma Technology) in order to clean and make the surfaces hydrophilic. Then 0.05 mL of the clean and concentrated gold particle solution was cast on the top of the template. The system was placed in a chamber with controlled humidity (99 %) until the particle sedimentation (24 h). After that, the system was removed from the humidity chamber and allowed to dry. This procedure allows for the direct stamping of pyramidal arrays on whatever surface including silicon wafers, glass, Tesafilm or cured PDMS.

Characterization. UV-VIS spectroscopy was recorded with a PerkinElmer, Lambda 19. Size, shape and topographical characterization of the nanoparticles and their assembles were characterized with transmission and scanning electron microscopy (TEM, LEO 922 EFTEM operating at 200 kV and LEO 1530 FE-SEM, Zeiss) and atomic force microscopy (NanoScope Dimension IIIm NanoScope V, Veeco Metrology Group). For the mass spectra, a Micromass® Quattro micro™ API was used and ions were generated using electrospray ionization (ESI) source, with a voltage of 5000 V (to optimize ionization efficiency) applied to the needle, and a cone voltage of 55 V. ^{1}H NMR spectra were recorded in $CDCl_3$ and CD_2Cl_2 at ambient temperature on a Bruker AMX-400 spectrometer at 400 MHz with residual protic solvent as the internal reference [$CDCl_3$, $\delta_H = 7.26$ ppm; CD_2Cl_2, $\delta_H = 5.32$ ppm]. ^{13}C NMR spectra were recorded in $CDCl_3$ and CD_2Cl_2 at ambient temperature on the same spectrometer at 100 MHz, with the

central peak of $CDCl_3$, δ_C = 77.16 ppm; CD_2Cl_2, δ_C = 54.0 ppm) as the internal reference. FTIR-ATR spectra were obtained on a JASCO IR 4200 spectrophotometer, with an ATR accessory (PIKE instruments) having a diamond ATR crystal. Raman and SERS were carried out with either a Renishaw Invia system or a handheld portable DeltaNu Raman Inspector instrument, exciting the sample with a 785 (diode) nm.

Theoretical calculations. Finite element method (FEM) electromagnetic simulations were performed with the COMSOL Multiphysics package using the RF module to completely solve the Maxwell equations. Gold spheres of 70 nm in diameter were considered, with the metal described through its measured dielectric function [4] and including a 0.5 nm coating of refractive index 1.3 to effectively represent the linking layer. The gold-to-gold gap distance was set to 1 nm in all cases. Excitation was carried out considering a monochromatic light (785 nm) parallel to the z axis.

SERS characterization of the supercrystals: In order to characterize the optical enhancing properties of the pyramidal supercrystals, a minute amount (10 μL) of a diluted solution (10^{-8} M) of 1-naphthalenethiol (1NAT, Aldrich) was spin-coated on 1 cm^2 of the pyramid film. Surfaces were then mapped using the Renishaw´s StreamLine accessory, taking mapping areas of 26 × 36 μm^2, with a step size of 500 nm (x100 objective) upon excitation with an NIR (785 nm) laser line. Acquisition times were set to 10 ms with power at the sample of 10 μW. The SERS response with the same analyte on other common substrates including evaporated gold and silver island films and aggregated gold and silver colloids can be found in ref. [5]. Intensity dependence with the morphology of the pyramid was studied point by point by with and step size of 500 nm and setting the autofocus track in a high confocality mode for each measurement. To probe single molecule detection, the same procedure was carried out using ethanolic solutions of crystal violet (10 μL, 10^{-12} M per cm^2) giving rise to an average concentration of less than 1 molecule/ μm (0.06 molecules/μm^2) [6]. The film was mapped using the Renishaw´s StreamLine accessory with a 100x objective (spatial resolution of 500 nm), acquisition times of 2 s and a power at the sample of 1 μW. Comparison between the Raman microscope and the handheld Raman system were

carried out on the samples prepared with 1 NAT by using, in the case of the Raman microscope, a macrosampling objective with an spatial resolution of 1 mm, equivalent to that of the portable system.

Phorphyrin synthesis: Solvents were dried according to published methods and distilled before use. All other reagents were commercial compounds of the highest purity available. Unless otherwise indicated all reactions were carried out under argon atmosphere in oven-dried glassware. Analytical thin layer chromatography (TLC) was performed on aluminium plates with Merck Kieselgel 60F254 and visualized by UV irradiation (254 nm) or by staining with an ethanolic solution of phosphomolibdic acid. Flash column chromatography was carried out using Merck Kieselgel 60 (230-400 mesh) under pressure.

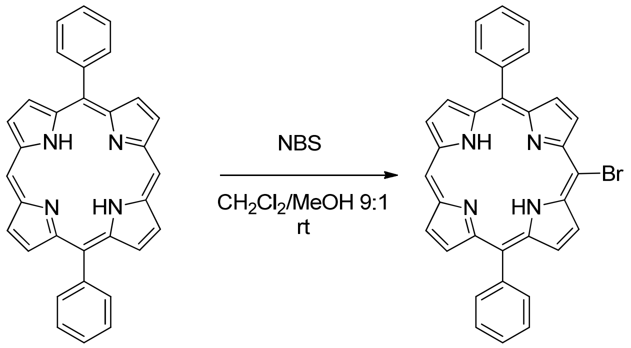

5-Bromo-10,20-diphenylporphyrin (BDPP) [7]: 5,15-Diphenylporphyrin (9.6 mg, 0.021 mmol) was dissolved in CH_2Cl_2/MeOH (9:1, 4.9 mL) and NBS (4.1 mg, 0.023 mmol) was added. The reaction mixture was stirred under air at room temperature for 15 min and quenched with acetone (1.0 mL). The solvent was evaporated. The residue obtained was purified by flash chromatography (silicagel, hexane/CH_2Cl_2, gradient from 20% to 40%) to provide 8.5 mg (75 %) of 5-bromo-10,20-diphenylporphyrin (BDPP). ^{1}H NMR (400 MHz, CD_2Cl_2): δ = 10.19 (s, 1H), 9.75 (d, J = 4.8 Hz, 2H), 9.31 (d, J = 4.6 Hz, 2H), 8.97 (d, J = 4.7 Hz, 4H), 8.22 (dd, J = 1.5, 7.5 Hz, 4H), 7.88 – 7.76 (m, 6H), -3.08 (s, 2H) ppm. ^{13}C NMR (100 MHz, CD_2Cl_2): δ = 141.8 (2x), 135.2 (4x), 132.9 (4x), 132.6 (4x), 132.3 (8x), 128.5 (2x), 127.5 (4x), 120.9 (2x), 106.1 (1x), 103.9 (1x) ppm. FTIR-ATR: ν = 2920 (s), 2851 (s), 1461 (m), 1260 (m) cm^{-1}. MS (ESI$^+$) m/z: 541.4 (90, $[M+H]^+$, ^{79}Br), 543.4 (100, $[M+H]^+$, ^{81}Br).

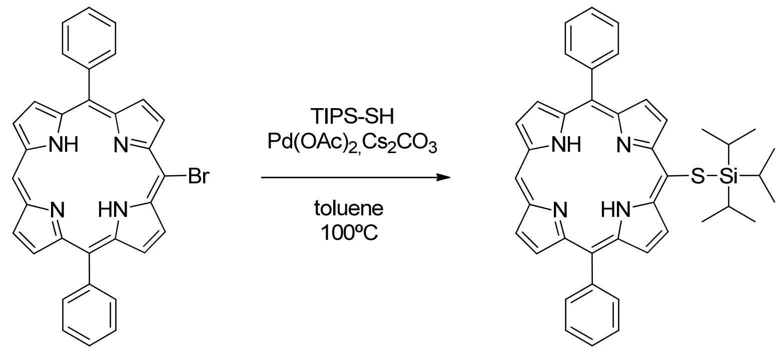

5-[(Triisopropylsilyl)thio]-10,20-diphenylporphyrin (TDPP) [8]: A sealable tube was charged with $Pd(OAc)_2$ (1.9 mg, 0.003 mmol), PPh_3 (3.1 mg, 0.012 mmol) Cs_2CO_3 (36.2 mg, 0.111 mmol), 5-bromo-10,20-diphenylporphyrin (30 mg, 0.055 mmol) and dry toluene (0.6 mL). The mixture was carefully degassed by freeze/thaw cycles (3x). Triisopropylsilanethiol (15.5 μL, 0.072 mmol) were added subsequently via syringe and the tube was sealed afterwards. The solution was warmed to 100 ^oC for 2 h. After cooling to room temperature, a saturated aqueous solution of NH_4Cl was added and the mixture was extracted with CH_2Cl_2 (3x). The collected organic layers were dried (Na_2SO_4) and filtered, and the solvent was evaporated. The crude was purified by flash chromatography (silicagel, gradient from 60:40 to 40:60 hexane/CH_2Cl_2) to obtain 13.1 mg (36%) of 5-[(triisopropylsilyl)thio]-10,20-diphenylporphyrin. ^1H NMR (400 MHz, $CDCl_3$): δ = 10.12 (s, 1H), 10.10 (d, *J* = 4.8 Hz, 2H), 9.27 (d, *J* = 4.6 Hz, 2H), 8.96 (d, *J* = 4.6 Hz, 2H), 8.93 (d, *J* = 11.2, 4.8 Hz, 2H), 8.31– 8.20 (m, 4H), 7.86 – 7.74 (m, 6H), 1.38 (h, *J* = 7.5 Hz, 3H), 0.89 (d, *J* = 7.5 Hz, 18H), -2.87 (s, 2H) ppm. ^{13}C NMR (100 MHz, $CDCl_3$): δ = 141.9 (2x), 134.8 (4x), 131.6 (4x), 131.3 (4x), 131.1 (8x), 127.9 (2x), 126.9 (4x), 119.9 (2x), 110.2 (1x), 105.3 (1x), 18.5 (6x), 13.9 (3x) ppm. FTIR-ATR: ν = 2924 (s), 2854 (s), 1465 (m), 1252 (m) cm^{-1}. MS (ESI^+) m/z: 987.8.4 (20, $[thiol-dimer +H]^+$), 493.5 (93, $[thiol-dimer +2H]^{2+}$)[9].

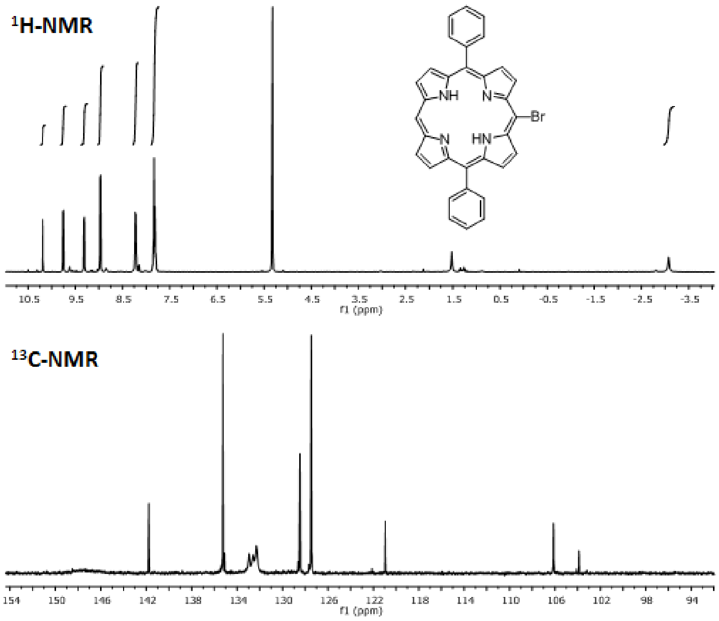

Fig. S1: ^{1}H and ^{13}C-NMR spectra for 5-Bromo-10,20-diphenylporphyrin (BDPP)

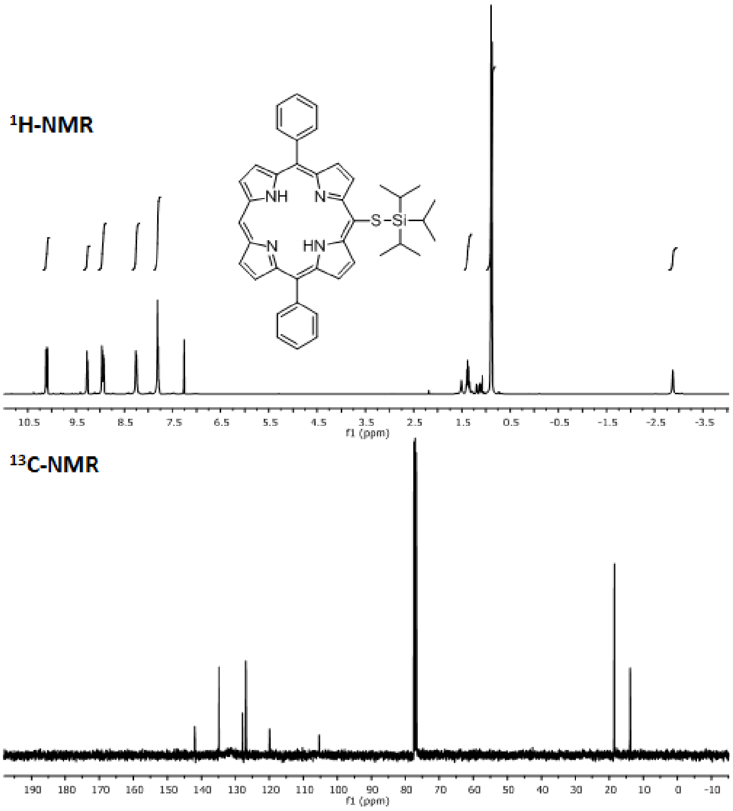

Fig. S2: ^{1}H and ^{13}C-NMR spectra for 5-[(Triisopropylsilyl)thio]-10,20-diphenylporphyrin (TDPP)

Supercrystal functionalization and sensing: The porphyrin was metallized by adding equimolecular concentrations of porphyrin and iron (II) acetate dihydrate to a solution of 5:1

dichloromethane/methanol. The solution was heated for 30 min and then allowed to stir overnight under a nitrogen atmosphere. Then, 10 µL of diluted solutions of TDPP-Fe ($3x10^{-5}$, $3x10^{-6}$, $3x10^{-7}$, $3x10^{-8}$, $3x10^{-9}$, $3x10^{-10}$, or $3x10^{-11}$ M) were cast on the pyramidal arrays (1 cm^2) and air dried. The biosensor mimic was evaluated against pure atmospheres of CO (Air Liquide) with the hand held Raman system to set the amount of TDPP-Fe necessary to quantitative detect the gas with detection ranges in between 1 to 400 ppm. The sensor was then evaluated in a close chamber containing normal atmosphere and controlling the amount of CO from a commercial mixture of CO (100 ppm) in N_2 (Airliquid) with the help of a manoreductor and a gas flow-meter. Reversibility studies were carried out on the same sample by exposure the sensor several times to the atmospheres containing 100 ppm of CO and clean air.

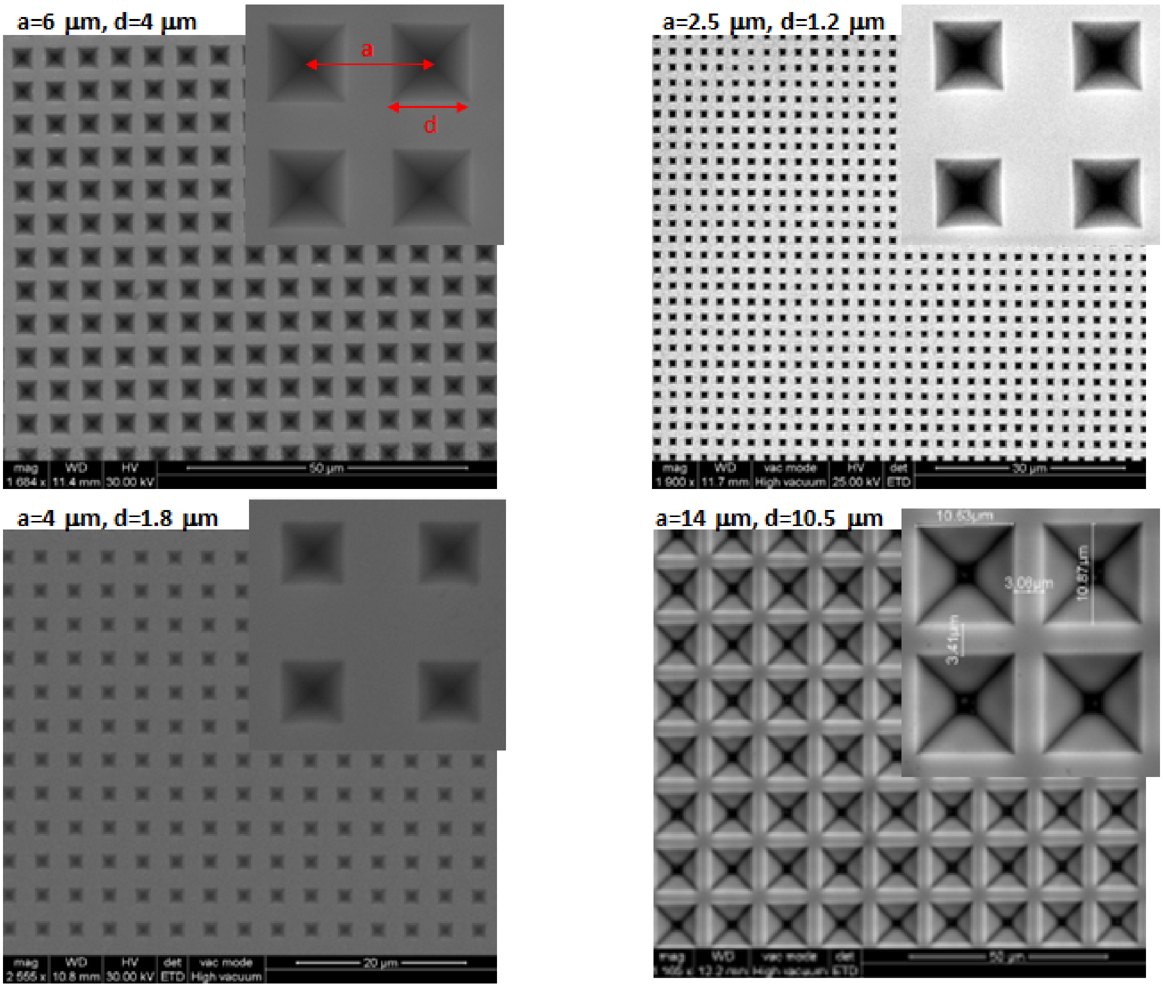

Fig. S3: Examples of other inverted pyramid templates obtained with direct laser writing lithography. Interestingly the resolution of the period and size can be controlled at the 100 nm.

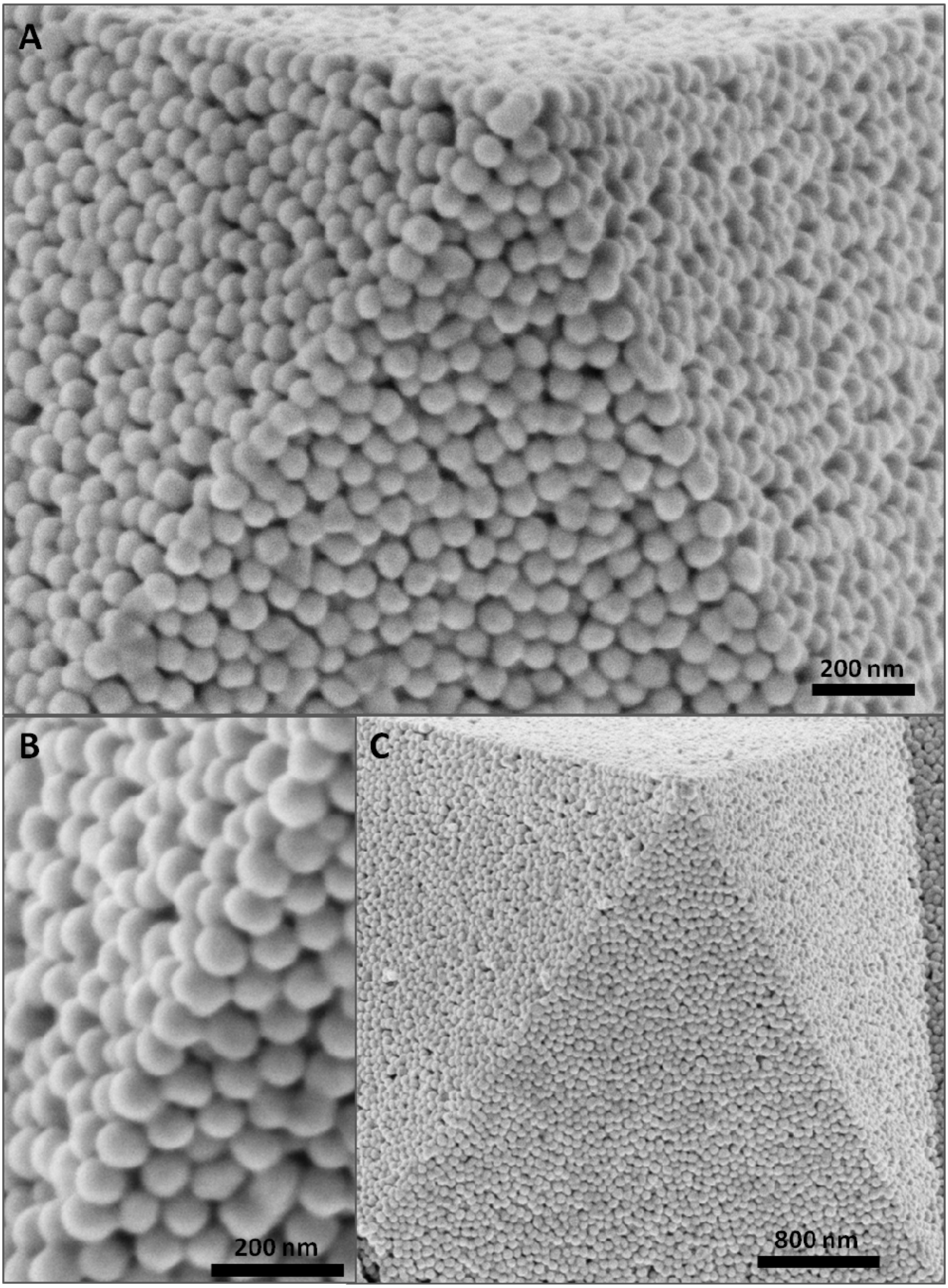

Fig. S4: High magnification SEM image of the tip (A), edge (B) and whole (C) of pyramidal structures (after (A) and before (B, C) plasma treatment) showing its nanostructure formed by individual gold nanoparticles.

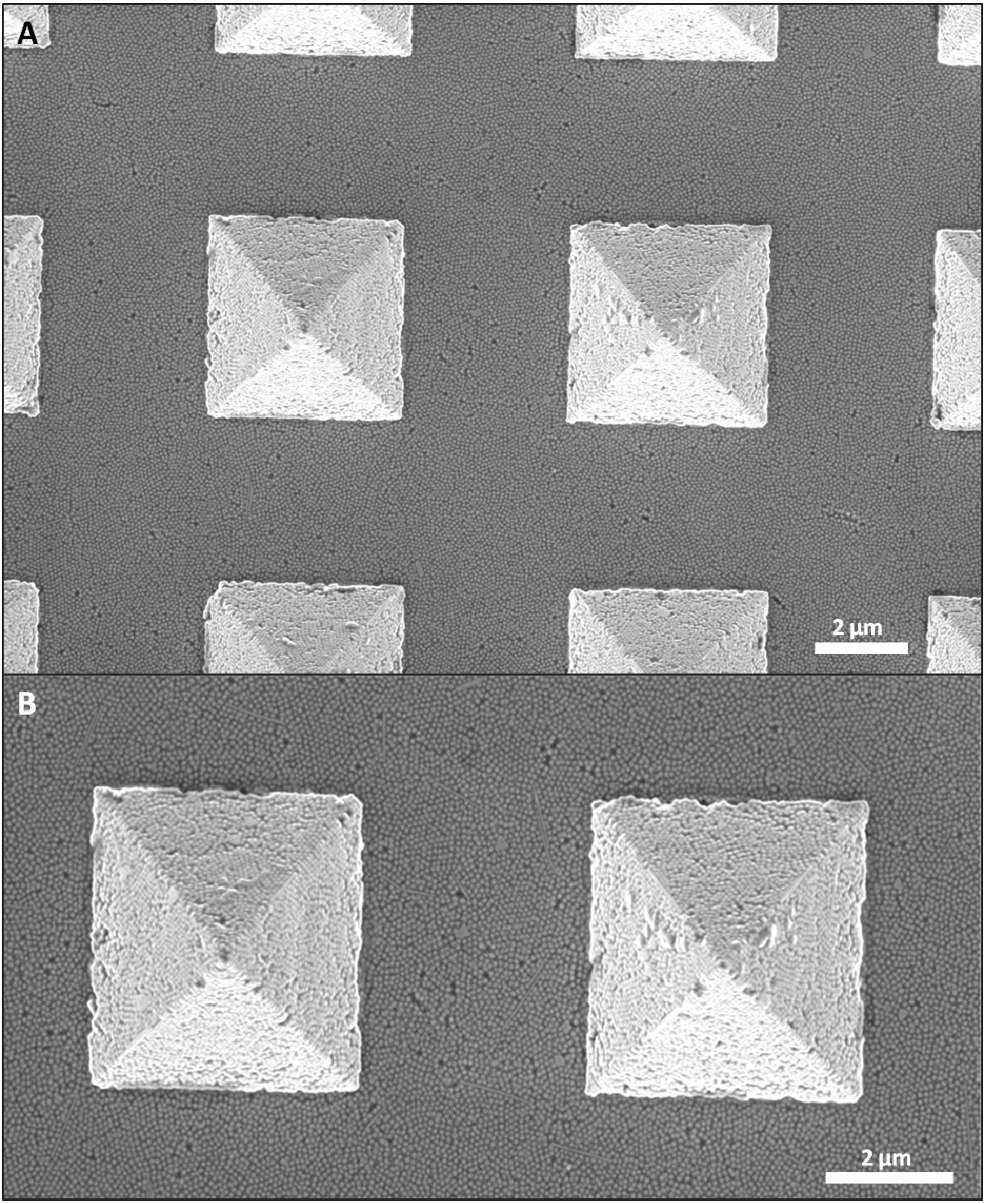

Fig. S5: Lower magnification SEM images (A, B) of various pyramidal structures still showing its nanostructure formed by individual gold nanoparticles and revealing the homogeneity of the assemblies.

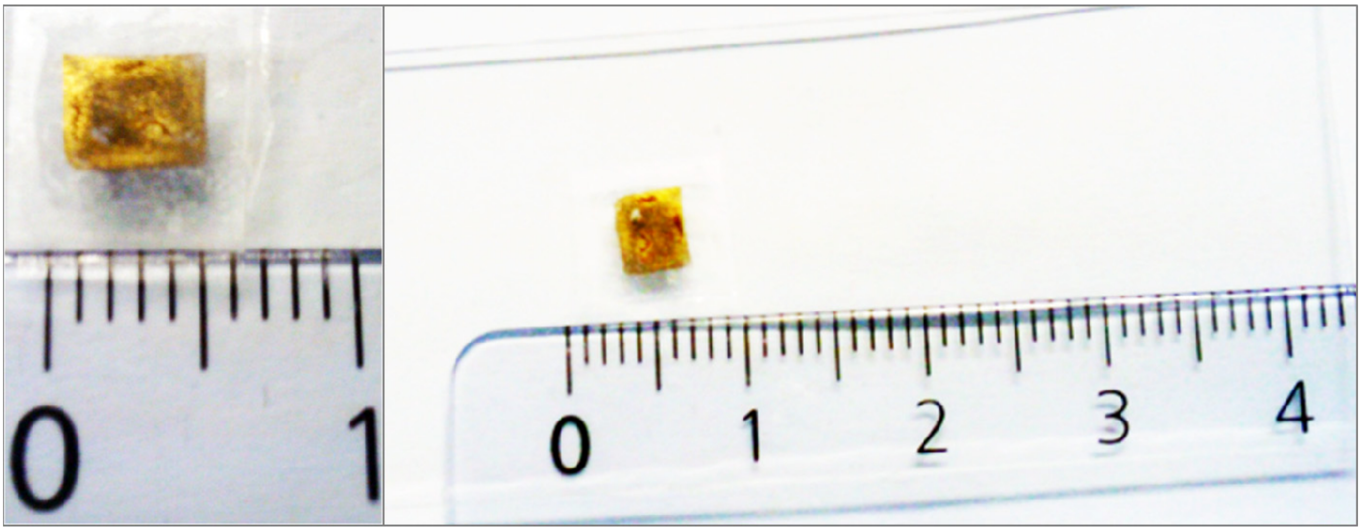

Fig. S6: Digital photograph images of macro-scale (0.5 X 0.4 cm) stamped substrates consisting of ordered micro-pyramidal structures of 4.4 µm side and 3.0 µm height formed with nanoparticles of 70 nm as building blocks.

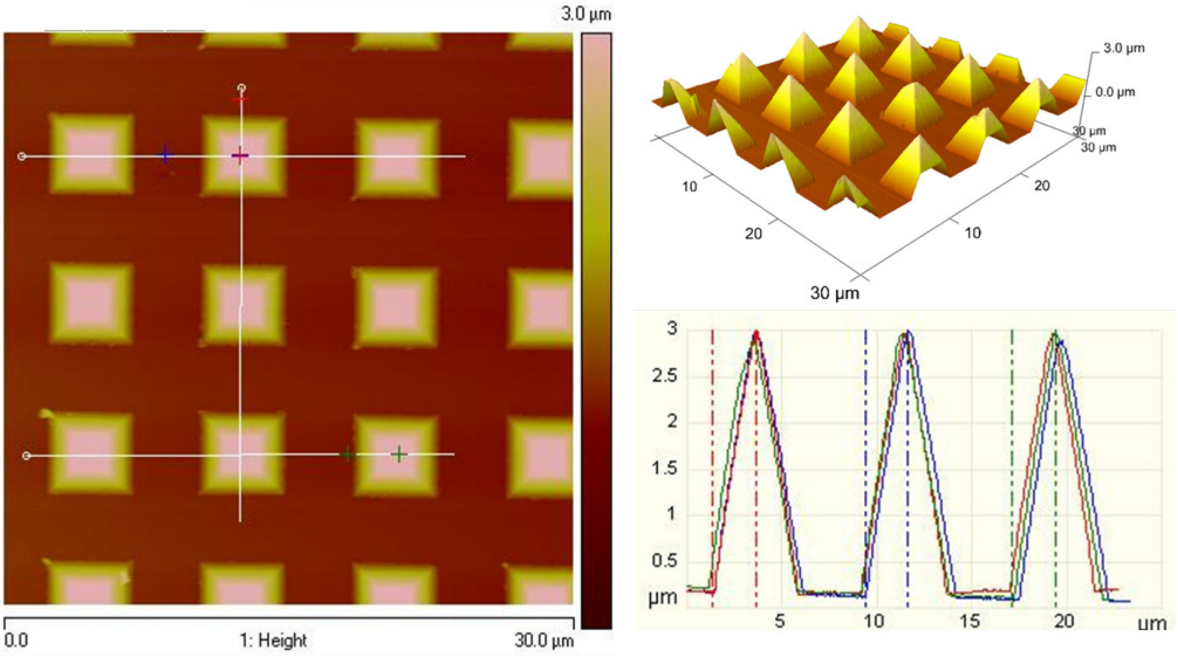

Fig. S7: Atomic force microscopy of the macroscaled nanostructured Au NP pyramid array film.

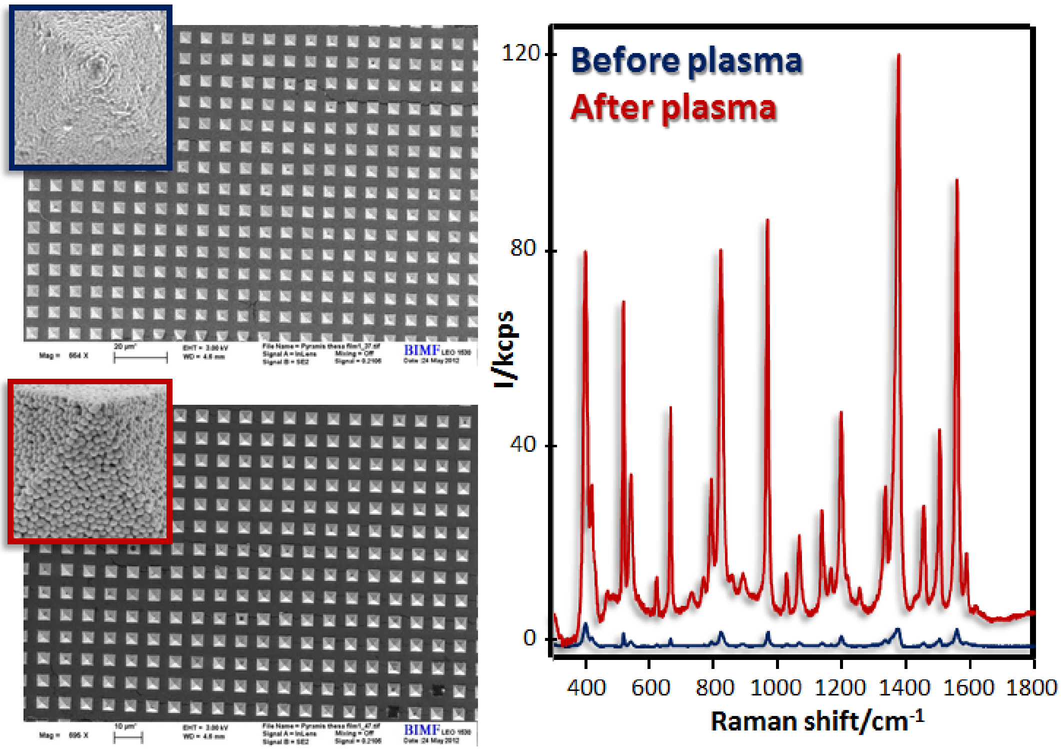

Fig. S8: SEM images and SERS spectra of macroscaled nanostructured Au NP pyramid array film before and after the plasma cleaning.

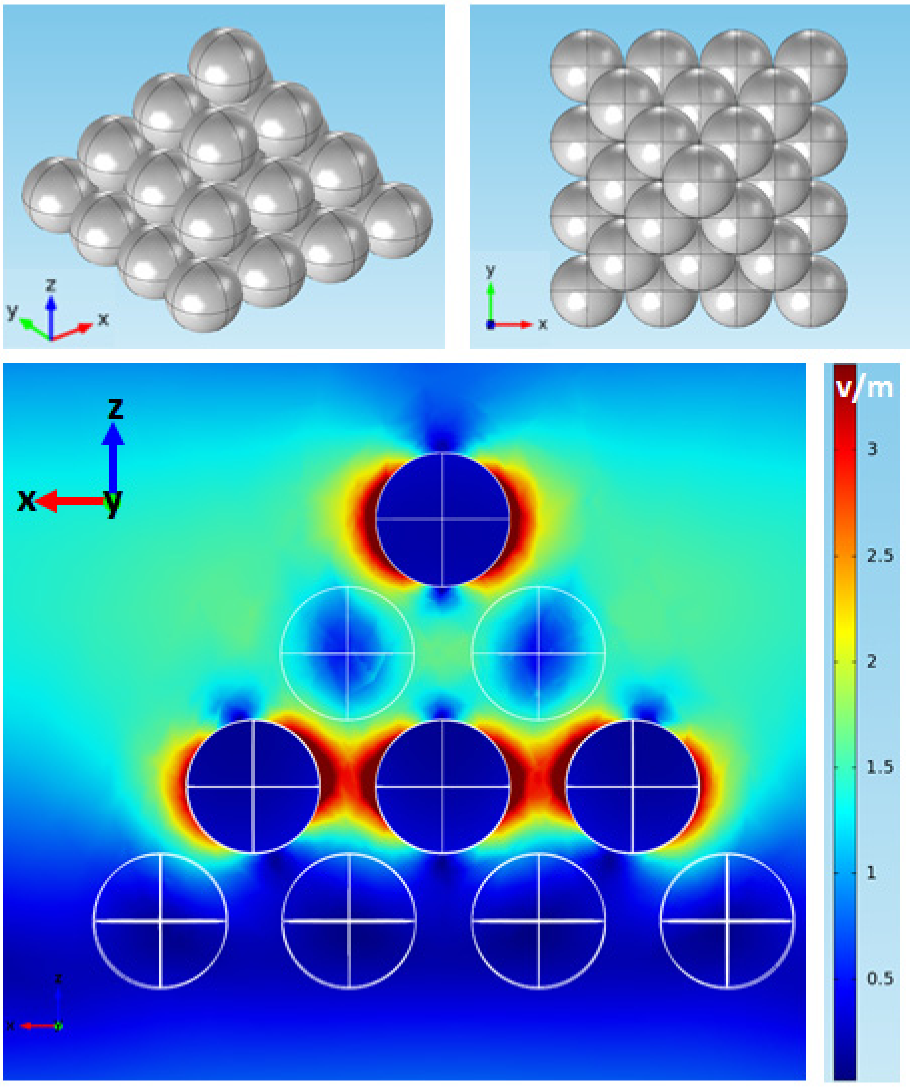

Fig. S9: Geometrical models used for the simulation and near-field electric distribution (V/m) upon excitation with 785 nm light.

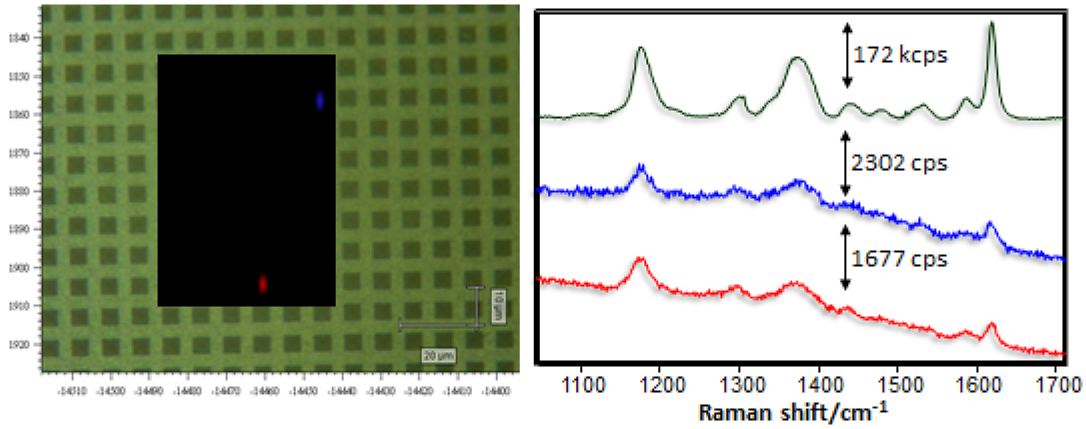

Fig. S10: Single molecule detection of crystal violet (CV) on the macroscaled nanostructured Au NP pyramid array film. Green spectrum represents the resembled SERS signal of CV; blue and red spectra correspond to the signal recorded at the blue and red spots in the Raman map. The CV film was prepared by spin-coating a minute volume (10 µL) of CV 10^{-12} M giving rise to an average concentration of less than 1 molecule/ µm (0.06 molecules/μm^2). The film was mapped using the Renishaw´s StreamLine accessory with a 100x objective (spatial resolution of 500 nm), acquisition times of 2 s and a power at the sample of 1 µW.

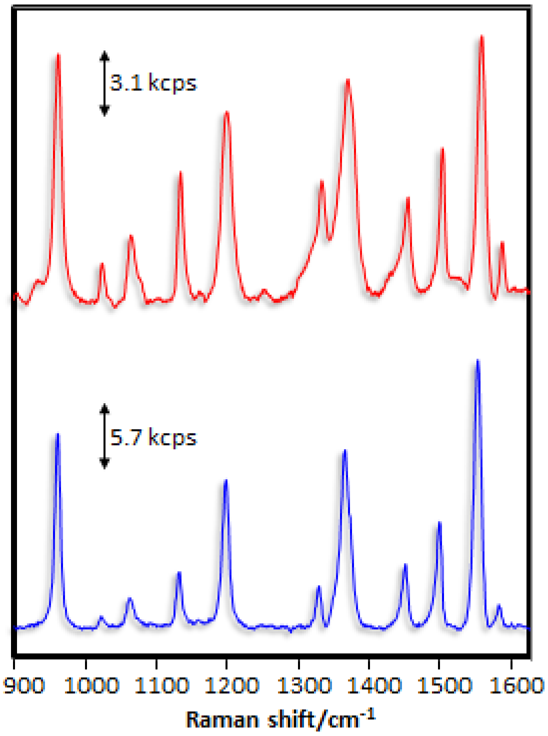

Fig. S11: Comparison of the SERS spectra of 1NAT as acquired with a portable handheld Raman system (red) or a bench microRaman instrument (using a macrosampling objective, W.D. 15 mm). Note that although both spectra fit band to band, for the one obtained with the portable the FHW is slightly larger as a consequence of the smaller focal length grating-detector.

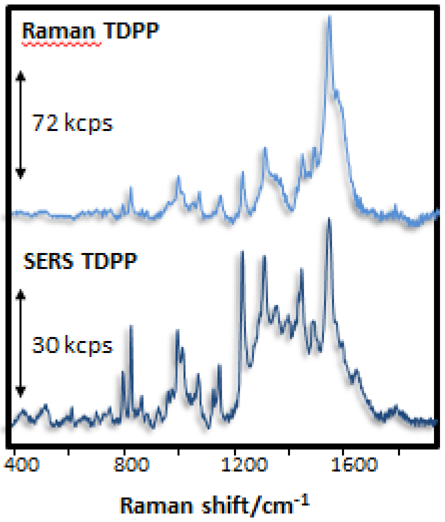

Fig. S12: Raman and SERS spectra of the TDPP. Both spectra fit band to band but with some differences in the relative intensity of some bands. Changes in relative intensity are due to the surface selection rules [10] and can be used to discern the orientation of the molecule at the metallic surface [11]. First, with the use of the 785 nm laser line excitation at the red of the bulk plasma resonance, the main component of the field at the surface is the normal to the surface. Considering the high affinity of the thiol and amino groups for gold, the adsorbed molecule could be either flat on the metal surface, interacting by porphyrin ring or with the molecular plane almost perpendicular to the metal surface if interacting through the terminal thiol group. The observed SERS bands can be safely assigned to ring deformations in the plane of the molecule. In fact, the SERS spectra contain all of the in-plane vibrational frequencies (ring stretching and C-H bending in the 1000-1650 cm^{-1} region) with remarkably strong relative intensity, as compared to those corresponding to out-of-plane modes. These latter observations discard the flat-on orientation and indicate that the ligand is bonded through the thiol group with its aromatic chromophore perpendicular to the gold surface, consistent with the higher reactivity toward gold of the thiols as compared with ternary amines.

References and Notes:

1. Pazos-Perez, N., Garcia de Abajo, F.J., Fery, A. & Alvarez-Puebla, R.A. From Nano to Micro: Synthesis and Optical Properties of Homogeneous Spheroidal Gold Particles and Their Superlattices. *Langmuir* **28**, 8909-8914 (2012).
2. Burda, C., Chen, X., Narayanan, R. & El-Sayed, M.A. Chemistry and Properties of Nanocrystals of Different Shapes. *Chem. Rev.* **105**, 1025-1102 (2005).
3. Orendorff, C.J., Sau, T.K. & Murphy, C.J. Shape-Dependent Plasmon-Resonant Gold Nanoparticles. *Small* **2**, 636-639 (2006).
4. Johnson, P.B. & Christy, R.W. Optical Constants of the Noble Metals. *Phys. Rev. B* **6**, 4370-4379 (1972).
5. Alvarez-Puebla, R.A., Dos Santos Jr, D.S. & Aroca, R.F. Surface-enhanced Raman scattering for ultrasensitive chemical analysis of 1 and 2-naphthalenethiols. *Analyst* **129**, 1251-1256 (2004).
6. Pieczonka, N.P.W. & Aroca, R.F. Single molecule analysis by surfaced-enhanced Raman scattering. *Chem. Soc. Rev.* **37**, 946-954 (2008).
7. Liu, C., Shen, D.-M. & Chen, Q.-Y. Unexpected bromination ring-opening of tetraarylporphyrins. *Chem. Comm.*, 770-772 (2006).
8. Kreis, M. & Bräse, S. A General and Efficient Method for the Synthesis of Silyl-Protected Arenethiols from Aryl Halides or Triflates. *Adv. Synth. Catal.* **347**, 313-319 (2005).
9. *Due to the instability of the protected thiol under the conditions of ESI-MS, only the product of deprotection and subsequent dimerization of the original thiol was observed by mass spectrometry.*
10. Moskovits, M. & Suh, J.S. Surface selection rules for surface-enhanced Raman spectroscopy: calculations and application to the surface-enhanced Raman spectrum of phthalazine on silver. *J. Phys. Chem.* **88**, 5526-5530 (1984).
11. *The orientation of molecules at metal surfaces can be inferred through the propensity toward enhancement of vibrational modes perpendicular to the surface. This propensity arises from the boundary condition which requires the electrostatic displacement, D, normal to the surface to be continuous across the interface:* $D_{\perp,in} = \varepsilon_{Au} E_{r=a}$; $D_{\perp,out} = \varepsilon_{surroundings} E_{r\neq a}$. *While the parallel component is simply:* $E_{\parallel,in,r\neq a} = E_{\parallel,out,r=a}$. *This leads to a preferential enhancement of the perpendicular electric field by a factor of* ε.
